# Assessment of hypoxia and its dynamic evolution in glioblastoma via qBOLD MRI: a comparative study with metformin treatment

**DOI:** 10.1186/s41747-024-00533-2

**Published:** 2024-12-02

**Authors:** Dongdong Wang, Jie Chen, Yinwei Ying, Xinxin Zhao, Nan Mei, Xuanxuan Li, Yuqi Zhu, Jin Cui, Pu-Yeh Wu, Yiping Lu, Bo Yin

**Affiliations:** 1 Department of Radiology, Huashan Hospital, Fudan University, Shanghai, China; 2 Department of Radiology, Ren Ji Hospital, School of Medicine, Shanghai Jiao Tong University, Shanghai, China; 3 GE Healthcare, Beijing, China

**Keywords:** Glioblastoma, Hypoxia, Magnetic resonance imaging, Metformin, Rats

## Abstract

**Background:**

To investigate the accuracy of quantitative blood oxygen level-dependent (qBOLD) magnetic resonance imaging (MRI) in identifying hypoxia within glioblastoma and explore dynamic changes in oxygenation status of glioblastoma with and without metformin administration.

**Methods:**

Three healthy and seven C6-bearing rats underwent 7-T qBOLD MRI. Oxygen extraction fraction (OEF) and cerebral metabolism rate of O_2_ (CMRO_2_) were calculated from qBOLD data. Tumor tissues were stained using hypoxia-inducible factor-1$\alpha$ (HIF-1$\alpha$) and pimonidazole. The correlation between the hypoxia markers and corresponding qBOLD-based parameters was analyzed. Six C6-bearing rats were divided into metformin-treated and control groups for a longitudinal study of qBOLD imaging changes, with scans conducted on the 12th, 15th, and 18th day post-tumor implantation.

**Results:**

In healthy rats, gray matter showed higher values than white matter in T2, T2*, cerebral blood volume (CBV), and cerebral blood flow (CBF), whereas OEF was lower. Glioblastoma tissues exhibited elevated T2, T2*, CBV, and CBF but decreased OEF and CMRO_2_ relative to normal-appearing white matter. No significant correlation was found between staining scores from HIF-1$\alpha$ and pimonidazole. T2* and T2 values were negatively correlated with pimonidazole scores in tumor regions. As the tumor progressed, OEF values increased with intra-tissue variations, whereas CMRO_2_ decreased. Metformin delayed the reduction of T2 and T2* values, with significant differences in OEF and CMRO_2_ values compared to controls on day 18.

**Conclusion:**

T2* and T2 values were significantly associated with the hypoxia status in glioma. Metformin could potentially mitigate the progression of hypoxia in glioblastoma, which can be tracked by qBOLD parameters.

**Relevance statement:**

This study demonstrates the potential of qBOLD parameters in assessing glioma dynamic oxygen metabolism and the efficacy of metformin as an anti-hypoxic agent, providing insights into improving glioblastoma treatment strategies.

**Key Points:**

- The study investigated qBOLD imaging’s accuracy in identifying hypoxia status within glioblastoma.
- qBOLD effectively assesses hypoxia and its dynamic evolution in glioblastoma.
- qBOLD parameters assist in identifying a suitable patient demographic for metformin treatment.

**Graphical Abstract:**

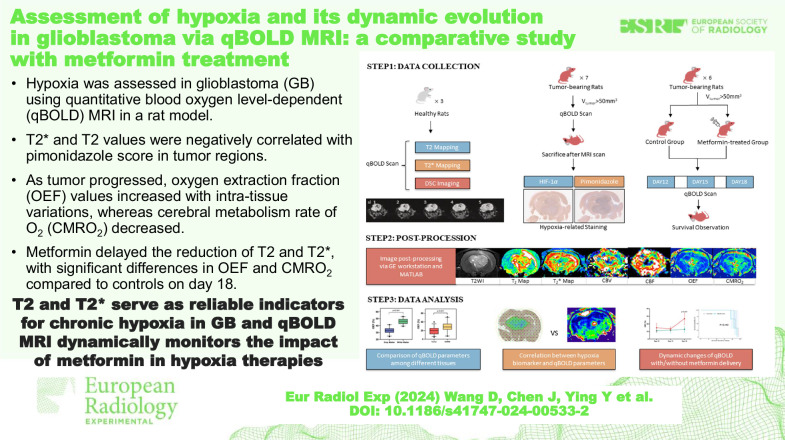

## Background

Glioblastoma (GB), the most common and aggressive primary brain tumor in adults, comprises approximately 15% of all brain tumors [1]. Despite standard treatments, the prognosis remains very poor, with median survival under two years [2]. The treatment challenges for GB are partially attributed to its highly hypoxic microenvironment, a result of the tumor’s aberrant microstructure. This hypoxic status, characterized by low oxygen tension (usually less than 10 mmHg), arises from a mismatch between the high oxygen consumption of rapidly proliferating tumor cells and the decreased oxygen supply due to dysfunctional neovascularization [3].

Hypoxic microenvironment in tumors promotes tumor growth, invasion, and resistance to chemo- and radio-therapy, making it a critical target for novel treatments [4]. However, the *in vivo* assessment of hypoxia in GB remains challenging, with techniques such as electron paramagnetic oximetry and ^18^F-FMISO positron emission tomography-computed tomography, being limited by their invasive and radiative nature [5, 6].

Recently, a noninvasive MRI technique named quantitative blood oxygenation level-dependent (qBOLD), which assesses magnetic susceptibility changes related to deoxygenated hemoglobin concentration, has been promising in the assessment of tumor hypoxic status [7]. It has been employed in both preclinical and clinical studies to assess oxygenation in GBs and evaluate therapeutic efficacy following treatments like anti-vascular endothelial growth factor drugs and carbogen inhalation [8–11]. However, qBOLD parameters’ accuracy in reflecting glioma hypoxia remains controversial [12–14]. And, no studies yet probe qBOLD-indicated oxygen metabolism dynamics in gliomas.

Metformin, a biguanide drug classically used to treat type 2 diabetes, has been recognized for its antineoplastic activity, with its usage associated with improved overall and progression-free survival in high-grade glioma patients [15]. Although the exact mechanism remains a topic of debate, several studies suggest that metformin’s anticancer effects might involve the down-regulation of hypoxia-inducible factor-1$\alpha$ (HIF-1α) expression and modulation of mitochondrial biogenesis [16–18].

Therefore, the present study aims to first validate the accuracy of the qBOLD technique for assessing tumor hypoxia in a malignant glioma-bearing rat model through correlation with pathological findings. Subsequently, we intend to explore the dynamic changes in tumor oxygenation status of glioma using qBOLD measurements with and without metformin administration. This could provide crucial insights into the clinical application of the qBOLD technique and the potential utility of metformin as a therapeutic agent in GB.

## Methods

All studies were approved by the Institutional Animal Care and Use Committee of the Medical School of Fudan University (2023-HSYY-539JZS).

### Rat model

A total of thirteen C6-bearing Wistar rats and three healthy Wistar rats were included in this study. These thirteen C6-bearing Wistar rats were randomly divided into two groups. The first group, consisting of seven C6-bearing Wistar rats and three healthy Wistar rats, underwent qBOLD MRI examination. Following the imaging, the seven C6-bearing Wistar rats were sacrificed for immunohistochemistry analysis. The remaining six C6-bearing Wistar rats making up the second group were further subdivided into a metformin administration group and a control group for a longitudinal study to assess the dynamic evolution of hypoxia using qBOLD imaging.

The detailed construction procedure for tumor-bearing rats was outlined in Supplemental File S1.

### MRI protocol

Seven days post-tumor implantation, MRI scanning was performed to assess the volume of each tumor twice a week. Rats were anesthetized with isoflurane provided by an ABS rodent anesthesia machine (Yuyan Instrument, Shanghai, China). MR imaging was conducted using a 7-T small-animal MRI scanner (Bruker PharmaScan, Ettlingen, Germany) equipped with a transmit-receive quadrature volume coil with an inner diameter of 3.8 cm. Coronal T1-weighted and T2-weighted imaging were performed. Then tumor volumes were calculated. All rats with tumor volumes reached 50 mm^3^ received the subsequent qBOLD MRI examination, which included three distinct sequences: T2 mapping, T2* mapping, and dynamic susceptibility contrast (DSC). The T2, T2* mapping, and DSC data were co-registered prior to postprocessing.

T2 mapping was conducted using a two-dimensional multiecho spin-echo sequence (repetition time 1,500 ms; echo time 12.5/25/37.5/50/62.5/75/87.5/100 ms; field of view 30 × 30 mm^2^; matrix size 256 × 256; slice thickness 1 mm; number of excitations 2). T2* mapping was executed with a three-dimensional multiecho gradient-echo sequence (repetition time 100 ms; echo time 3/9/15/21/27/33 ms; field of view 30 × 30 mm^2^; matrix size 256 × 256; slice thickness 1 mm; number of excitations 2). The DSC MRI was performed using a gradient-echo echo-planar imaging sequence (repetition time 400 ms; echo time 7.3 ms; field of view 30 × 30 mm^2^; matrix size 128 × 128; slice thickness 2 mm) with an injection of a suspension of 0.6 mL Gd-DTPA (Magnevist, Schering, Berlin, Germany) followed by 0.5 mL NaCl in a high-pressure syringe into the tail vein with a flow rate of 2 mL/s after the initial ten phases. The total scan time of qBOLD was approximately 18 min. More detailed parameters for the above-mentioned sequences and the specifics regarding the postprocessing of qBOLD data were listed in Supplemental File S2.

Six C6-bearing rats in the second group with tumor volume exceeding 50 mm^3^ were selected for further dynamic imaging observation. Starting from the 12th day post-tumor transplantation, three rats in the treatment group received daily peritoneal injections with metformin (30 mg/kg/day). The objective was to assess the natural progression of glioma and the anti-hypoxic effect of metformin. qBOLD scans were conducted on the 12th, 15th, and 18th day post-transplantation for both treatment and control groups. These six rats died from tumors and the overall survival periods were documented.

### Immunohistochemistry

Seven C6-bearing rats for an immunohistochemistry study received intravenous injections of pimonidazole HCl solution (60 mg/kg body weight) in phosphate-buffered saline buffer (Sigma, St. Louis, MO, USA) following qBOLD imaging. Phosphate-buffered saline is a buffer solution that mimics the ion concentration, osmolarity, and pH of human body fluids. One hour after injection, the rats were sacrificed. Overdoses of ketamine and medetomidine were administered prior to transcardial perfusion with 4% paraformaldehyde in phosphate-buffered saline. Subsequently, the brains were extracted, postfixed in 4% paraformaldehyde in phosphate-buffered saline overnight, dissected, and embedded in paraffin. Brain sections of five-micrometer thickness were obtained using a sliding microtome and processed.

We identified sections displaying consistent pathological structures corresponding to the largest coronal tumoral regions on T2-weighted imaging. These pathological sections were further cut into 4-μm slices for the following stains: hematoxylin and eosin staining, HIF-1α staining, and pimonidazole Staining. The results were examined under a microscope and recorded with imaging software (DP80, Olympus, Tokyo, Japan) and subsequently archived in whole slide image format for the following analysis.

### Antibodies used for histology analysis

HIF-1α staining was performed using a rabbit monoclonal anti-human HIF-1a antibody (1:50, ab51608, Abcam, Cambridge, UK) with a 16-h incubation at 4 °C. Anti-pimonidazole mouse IgG1 monoclonal antibody (MAb1, Lot # 9.7.11, 60 μg/mL) was used for pimonidazole staining.

### MRI and pathological analysis

Quantitative analysis of HIF-1α and pimonidazole was quantitively evaluated by HALO image analysis software (Indica Labs, Albuquerque, NM, USA) [19]. All regions of interest (ROIs) were 1 mm^2^ in area. The HIF-1α/pimonidazole scores were calculated by multiplying the scores of staining intensity and percentage of positive cells. The inclusion criteria for ROIs were: (a) containing only tumor or normal tissue and (b) having less than 25% of components including vessels, necrosis or calcification, and without air. ROIs within 1 mm from the edge of the tumor were classified as peripheral intratumoral parts, otherwise as central intratumoral parts.

An in-house MATLAB (Mathworks, Natick, MA, USA) script was used for quantitative analysis. T2, T2*, CBF, CBV, OEF, and CMRO_2_ maps were subdivided into multiple ROIs corresponding to the subdivision of the corresponding pathological slices. Imaging parameters of all areas were automatically output for statistical analyses.

### Data analysis

For imaging normal rats, the coronal T2-weighted image at the striatum level was selected. A senior neuroradiologist (with over 10 years of experience) manually delineated five 10-mm^2^ ROIs within the bilateral striatum and internal capsule (ten ROIs in total). For tumor-bearing rats, a coronal T2-weighted image with the largest tumor region was selected. The neuroradiologist manually drew five ROIs in the tumor region and five in normal tissue of the bilateral striatum. For dynamic imaging, the T2-weighted image with the largest tumor region was used to define a single ROI encompassing the tumor. These ROIs were then replicated onto corresponding T2, T2*, CBF, CBV, OEF, and CMRO_2_ maps for quantitative assessment.

Exclusion criteria: scans with artifacts not allowing a reliable assessment; scans with abnormal perfusion curves on DSC imaging, indicating unsuccessful acquisition; ROIs with T2, T2*, CBF, and CBV values below zero; and OEF values below 0% or over 100%.

**Fig. 1:**
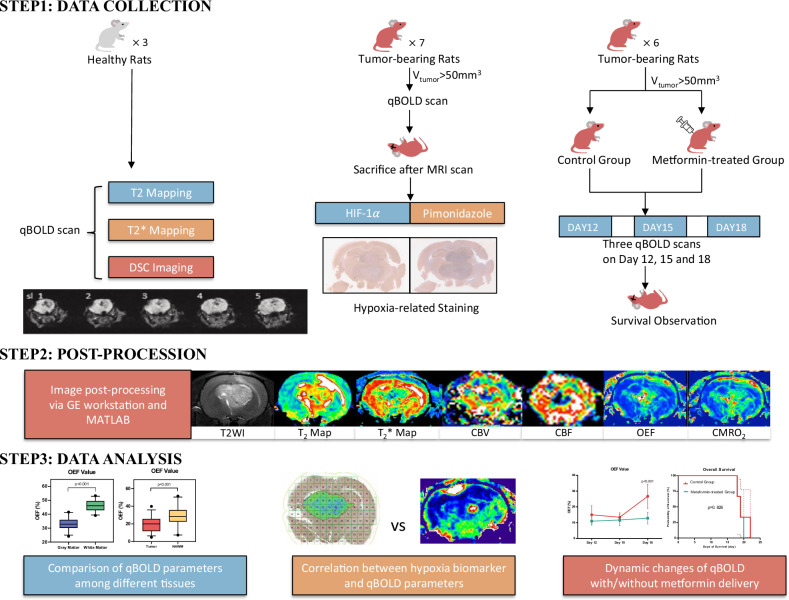
The workflow of the study

The basic workflow of our study is illustrated in Fig. 1.

### Statistical analysis

All statistical analyses were performed using SPSS software (version 20.0, IBM Corp, Armonk, NY, USA). Continuous variables between groups were compared using a two-sided unpaired *t*-test, whereas discrete variables between groups were analyzed by χ^2^ test and Fisher’s exact test. Normally distributed quantitative data were expressed as mean ± standard deviation, while categorical data were presented as numbers and percentages. The assumption of normality for the data was verified using the Shapiro–Wilk test. A two-tailed *p*-value less than 0.05 was considered statistically significant.

## Results

A total of 16 rats (3 healthy rats and 13 C6-bearing rats) were included in this study.

### qBOLD-based O_2_ metabolism in healthy rats

**Fig. 2:**
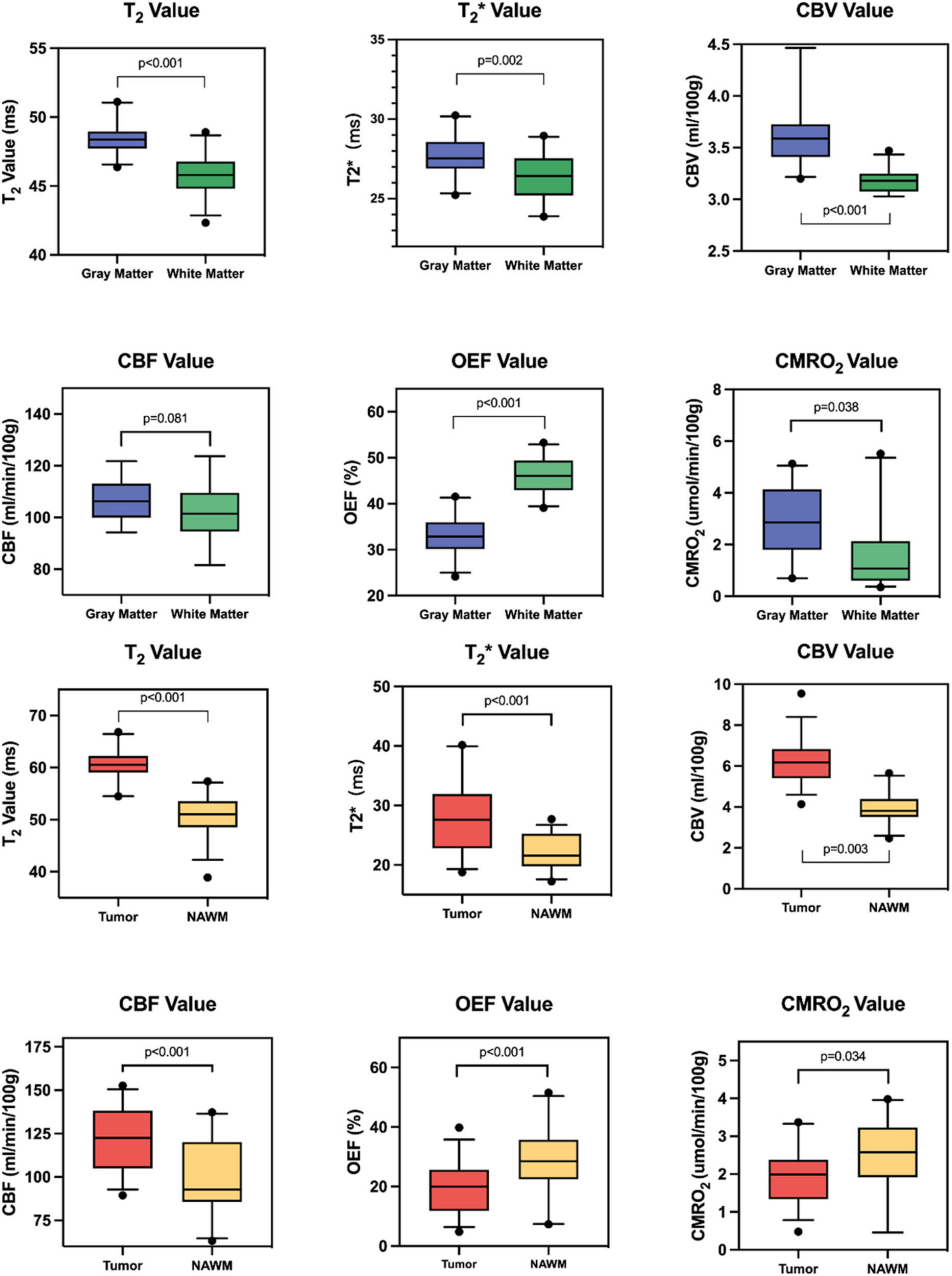
Differences in qBOLD parameters between GM and WM in healthy rats, and between tumor tissue and contralateral NAWM in C6-bearing rats. CBF, Cerebral blood flow; CBV, Cerebral blood volume; CMRO_2_, Cerebral metabolism rate of O_2_; OEF, Oxygen extraction fraction; qBOLD, Quantitative blood oxygen level-dependent

QBOLD parameters in gray matter (GM) in the striatum and white matter (WM) in the internal capsule showed notable distinctions. The global T2 and T2* relaxation times were significantly greater in GM compared to WM (T2 values: 48.5 ± 1.1 *versus* 45.8 ± 1.4 ms, *p* < 0.001; T2* values: 37.7 ± 1.3 *versus* 36.4 ± 1.5 ms, *p* = 0.002), suggesting differences in their relaxation properties. Cerebral blood volume (CBV) was also higher in GM (*p* < 0.001), but differences in cerebral blood flow (CBF) did not achieve statistical significance. Oxygen extraction fraction (OEF) was higher in WM, whereas the cerebral metabolic rate of oxygen (CMRO_2_) was significantly greater in GM, highlighting metabolic distinctions (Table 1 and Fig. 2). No significant interhemispheric differences in these parameters were noted.

**Table 1:** Differences in qBOLD parameters between GM and WM in healthy rats

qBOLD parameter	GM (mean ± SD)	WM (mean ± SD)	*p*-value
T2, (ms)	48.5 ± 1.1	45.8 ± 1.4	< 0.001
T2*, (ms)	27.7 ± 1.3	26.4 ± 1.5	0.002
CBV, (mL/100 g)	3.6 ± 0.3	3.2 ± 0.1	< 0.001
CBF, (mL/min/100 g)	107.0 ± 8.1	102.2 ± 10.8	0.081
OEF, (%)	33.1 ± 4.3	46.2 ± 3.9	< 0.001
CMRO_2_, (μmol/100 g/min)	3.6 ± 0.8	3.1 ± 1.0	0.038

*CBF* Cerebral blood flow, *CBV* Cerebral blood volume, *CMRO*_*2*_ Cerebral metabolism rate of O_2_, *NAWM* Normal-appearing white matter, *OEF* Oxygen extraction fraction, *qBOLD* Quantitative blood oxygen level-dependent, *SD* Standard deviation

### qBOLD-based O_2_ metabolism in C6-bearing rats

**Fig. 3:**
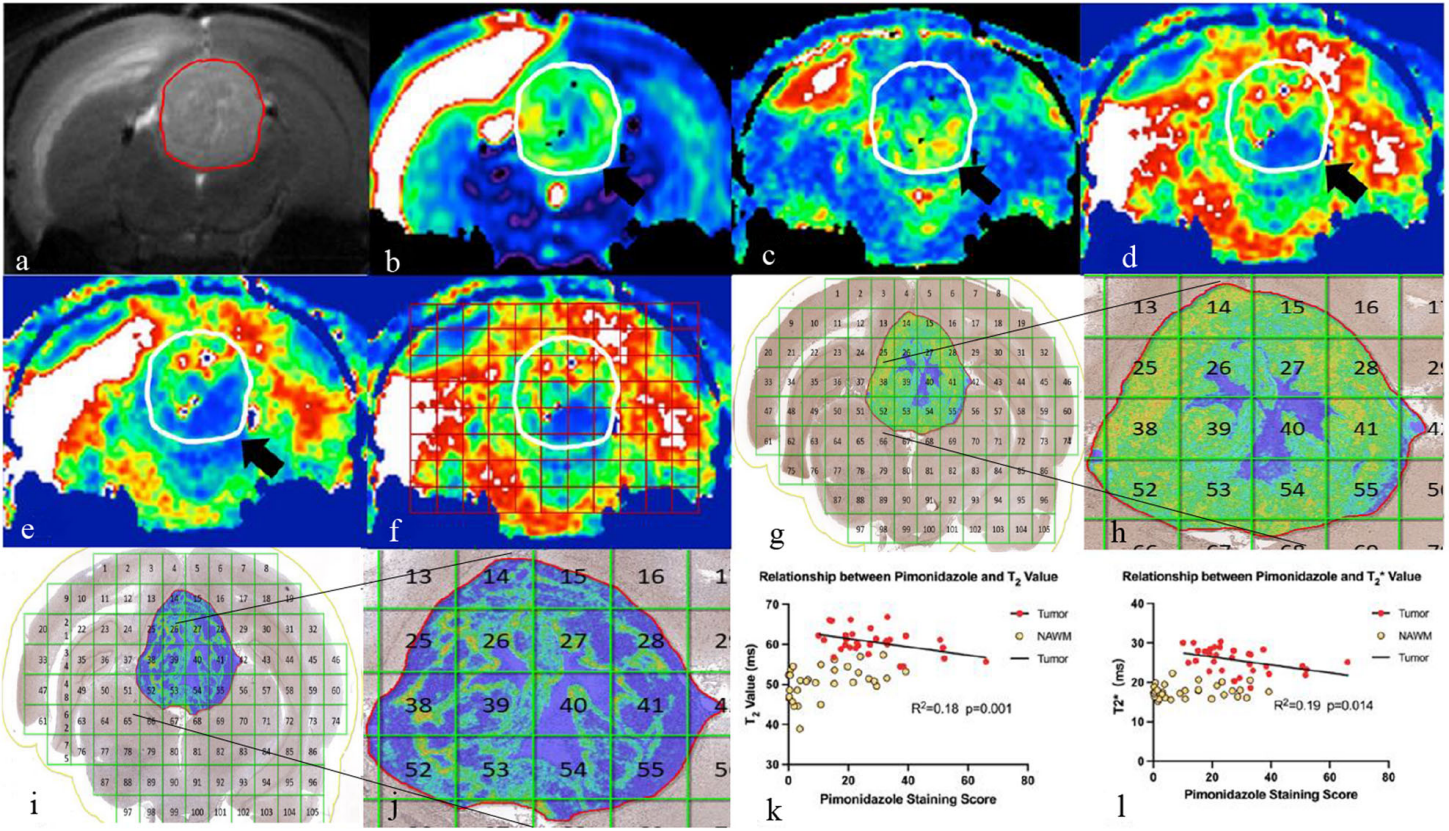
The correlation between qBOLD parameters and hypoxia biomarkers. **a** T2-weighted image of the tumor on the coronal plane. **b** T2 map of the tumor, in which the tumor was cycled in white. **c** T2* map of the tumor, in which the tumor was cycled in white. **d** qBOLD-based OEF map of the tumor, in which the tumor was cycled in white. **e** qBOLD-based CMRO_2_ map of the tumor, in which the tumor was cycled in white. **f** The CMRO_2_ map calculated by MATLAB for region-of-interest selection. **g** HIF-1α staining result produced by HALO software with the same region-of-interest distribution referred to qBOLD maps. **h** Magnification of the tumor region on HIF-1α staining result. **i** Pimonidazole staining result produced by HALO software. **j** Magnification of the tumor region on pimonidazole result. **k** Correlation between T2 value and pimonidazole staining score in the tumor region. **l** Correlation between T2* value and pimonidazole staining score in the tumor region. CMRO_2_, Cerebral metabolism rate of O_2_; OEF, Oxygen extraction fraction; HIF-1α, Hypoxia-inducible factor-1$\alpha$; qBOLD, Quantitative blood oxygen level-dependent

Compared to contralateral normal-appearing white matter (NAWM), tumor tissue exhibited significant differences across all qBOLD parameters, with higher T2, T2*, CBV, and CBF and lower OEF and CMRO_2_ (all *p*-values < 0.05, Table 2 and Fig. 3). Compared to WM in healthy controls, NAWM in C6-bearing rats exhibited reduced T2*, CBF, OEF and increased CMRO_2_ (all *p* < 0.001), indicating significant metabolic differences (Table 3).

**Table 2:** Differences in qBOLD parameters between tumor tissue and contralateral NAWM in C6-bearing rats

qBOLD parameter	NAWM (mean ± SD)	Tumor tissue (mean ± SD)	*p*-value
T2, (ms)	50.6 ± 4.1	60.6 ± 3.2	< 0.001
T2*, (ms)	22.4 ± 2.9	27.8 ± 5.7	< 0.001
CBV, (mL/100 g)	4.0 ± 0.8	6.2 ± 1.1	< 0.001
CBF, (mL/min/100 g)	99.5 ± 22.8	120.7 ± 18.5	< 0.001
OEF, (%)	28.7 ± 12.0	19.8 ± 8.7	< 0.001
CMRO_2_, (μmol/100 g/min)	2.4 ± 1.0	2.0 ± 0.8	0.033

*CBF* Cerebral blood flow, *CBV* Cerebral blood volume, *CMRO*_*2*_ Cerebral metabolism rate of O_2_, *NAWM* Normal-appearing white matter, *OEF* Oxygen extraction fraction, *qBOLD* Quantitative blood oxygen level-dependent, *SD* Standard deviation

**Table 3:** Differences in qBOLD parameters between WM in healthy rats and contralateral NAWM in C6-bearing rats

qBOLD parameter	WM in healthy rats, (mean ± SD)	WM in tumor-bearing rats, (mean ± SD)	*p*-value
T2, (ms)	45.8 ± 1.4	50.63 ± 4.1	< 0.001
T2*, (ms)	27.1 ± 1.5	22.4 ± 2.9	< 0.001
CBV, (mL/100 g)	3.2 ± 0.1	4.0 ± 0.8	< 0.001
CBF, (mL/min/100 g)	104.6 ± 9.8	99.5 ± 22.8	0.167
OEF, (%)	39.7 ± 7.8	28.7 ± 12.0	< 0.001
CMRO_2_, (μmol/100 g/min)	2.2 ± 1.5	2.4 ± 1.0	0.502

*CBF* Cerebral blood flow, *CBV* Cerebral blood volume, *CMRO*_*2*_ Cerebral metabolism rate of O_2_, *NAWM* Normal-appearing white matter, *OEF* Oxygen extraction fraction, *qBOLD* Quantitative blood oxygen level-dependent, *SD* Standard deviation

### Relationship between qBOLD parameters and hypoxia-related pathological staining

Differently from NAWM regions, tumor tissue exhibited remarkably high staining scores in both HIF-1a and pimonidazole, however, the HIF-1a and pimonidazole staining scores within the same tumor ROIs did not show a significant correlation (*p* = 0.111). T2* values (*r* = 0.44, *p* = 0.014) and T2 values (*r* = 0.43, *p* = 0.017) demonstrated significant negative relationships with the expression scores of pimonidazole in tumor regions (Fig. 3). However, no qBOLD parameters exhibited close relationships with HIF-1 expression.

### Dynamic changes of qBOLD parameters in tumors with/without metformin delivery

No significant differences were observed in either tumor size or overall survival between the control group and the metformin-treated group (all *p* > 0.05, Table 4).

**Table 4:** Differences in tumor volume and overall survival between metformin-treated and control groups

		Metformin-treated group	Control group	*p*-value
Tumor volume (mm^3^) after C6 cell implantation	Day 12	150.3 ± 8.5	142.8 ± 19.3	0.571
	Day 15	284.4 ± 38.3	245.1 ± 31.3	0.271
	Day 18	436.8 ± 96.8	375.2 ± 77.7	0.438
Overall survival (days)		19.67 (18.5–20.5)	19.0 (18.5–19.5)	0.826

Data are given as mean ± standard deviation or median (interquartile range)

**Fig. 4:**
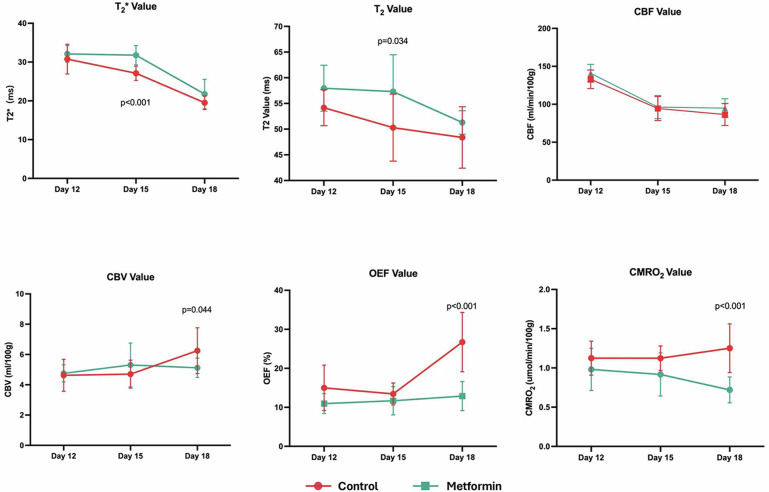
Comparison of qBOLD parameters between the metformin-treated group and control group on the 12th, 15th, and 18th day after tumor transplantation. CBF, Cerebral blood flow; CBV, Cerebral blood volume; CMRO_2_, Cerebral metabolism rate of O_2_; OEF, Oxygen extraction fraction; qBOLD, Quantitative blood oxygen level-dependent

**Fig. 5:**
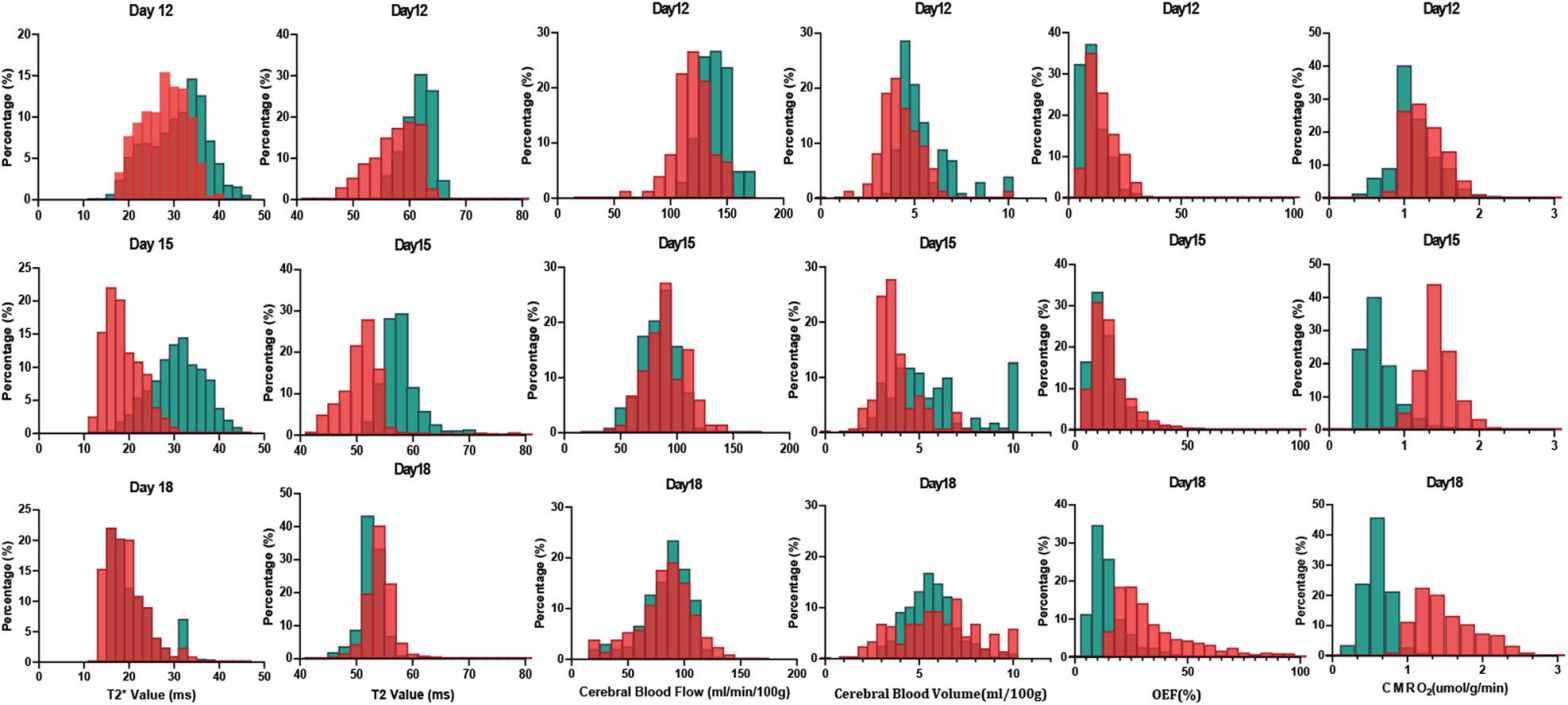
Histograms of qBOLD parameters in the metformin-treated group and control group on the 12th, 15th, and 18th day after tumor transplantation. CBF, Cerebral blood flow; CBV, Cerebral blood volume; CMRO_2_, Cerebral metabolism rate of O_2_; OEF, Oxygen extraction fraction; qBOLD, Quantitative blood oxygen level-dependent

**Fig. 6:**
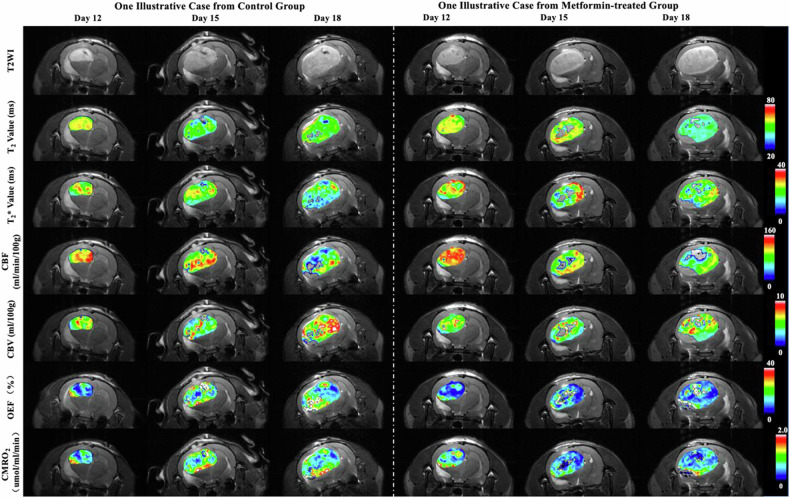
Illustration of the changes in qBOLD parameters in typical cases with and without metformin delivery. On day 15, T2 and T2* values remained at high levels in a metformin-treated group compared with the ones in the control group. Low levels of OEF and CMRO_2_ values were observed in the metformin-treated group. CBF, Cerebral blood flow; CBV, Cerebral blood volume; CMRO_2_, Cerebral metabolism rate of O_2_; OEF, Oxygen extraction fraction; qBOLD, Quantitative blood oxygen level-dependent

In the control group, as the tumor progressed, T2, T2*, and CBV values within the tumor tended to gradually decrease, accompanied by increasing unevenness in CBV distribution. OEF values in the tumor tissue gradually increased and showed large intra-tissue variations, while CMRO_2_ showed a declining trend (Figs. 4 and 5). For the metformin group, no significant differences were observed in the qBOLD parameters in the tumor compared to the control group on day 12 (before treatment). Three days after metformin injection (day 15), T2 and T2* values in C6 gliomas were significantly higher than those in the control group (T2 values: 57.3 ± 7.2 ms *versus* 50.3 ± 6.5 ms, *p* = 0.034; T2* values: 31.8 ± 2.5 ms *versus* 27.1 ± 1.9 ms, *p* < 0.001), but there was no significant difference between the two groups in T2 and T2* values on day 18. There were no significant differences in the dynamic changes of CBF and CBV within the tumor between the two groups. After metformin treatment, OEF values in the tumor regions remained at a lower level, and CMRO_2_ showed a gradually declining change, both of which were significantly different from the values in the control group on day 18 (OEF: 12.9 ± 3.7% *versus* 26.7 ± 7.6, *p* < 0.001; CMRO_2_: 0.7 ± 0.2 *versus* 1.3 ± 0.3 μmol/min/100 g, *p* < 0.001, Figs. 4 and 5). An illustration of the changes in qBOLD parameters in cases with and without metformin delivery is depicted in Fig. 6.

## Discussion

This study endeavors to demonstrate alterations in oxygen metabolism during tumor progression by evaluating the variances in qBOLD parameters within gliomas across both healthy and glioma-bearing models. It delves into the correlation between qBOLD parameters within gliomas and prevalent hypoxia markers, explores the longitudinal alterations in qBOLD parameters concurrent with tumor progression, and appraises the influence of metformin—a candidate with the potential to modify oxygen metabolism levels—on the oxygen metabolic landscape of gliomas.

In this study, GM demonstrated notably higher T2, T2*, CBF, and CBV values than WM, reflecting greater water content and vascular supply, with lower OEF but higher CMRO_2_, suggesting active metabolism and ample oxygen supply in GM. Conversely, tumor tissues exhibited similar physical characteristics to GM but had lower OEF and CMRO_2_ than NAWM in the contralateral hemisphere, indicating a rich blood supply but inefficient metabolic efficiency, potentially due to a preference for aerobic glycolysis in tumors [20].

This study innovated by integrating image quantification with hypoxia markers [12, 13, 21]. Our findings show no significant correlation between HIF-1α and pimonidazole expressions, and also no link between HIF-1α expression and qBOLD parameters. However, a significant negative correlation was observed between pimonidazole expression and T2 and T2* values, suggesting that lower T2 and T2* values in tumors are associated with higher pimonidazole levels, indicating increased hypoxia.

HIF-1α and pimonidazole serve as pivotal markers for tumor hypoxia, delineating acute and chronic hypoxic conditions, respectively [22]. In the tumor’s early stages, acute cyclic hypoxia prevails, stemming from vascular disruptions and blood supply deficits, temporarily elevating HIF-1α. As the tumor expands, it shifts to chronic hypoxia caused by diffusion-related oxygen deprivation.

Tumor growth often leads to disordered vasculature and excessive oxygen consumption by tumor cells, contributing to two main types of hypoxia: perfusion-related (acute) and diffusion-related (chronic) hypoxia [23]. Perfusion-related hypoxia arises from irregular blood flow within the tumor, caused by functional and structural deficiencies of the neovasculature. This form of hypoxia is typically transient, resulting from temporary blockages that elevate interstitial pressure and affect all tumor cells, irrespective of their distance from blood vessels. Diffusion-related hypoxia, on the other hand, occurs due to limitations in the diffusion of oxygen. It primarily affects cells that are situated farther from the nearest capillaries, typically within a range of 100–180 μm. As the tumor expands, the distance between these cells and the nearest oxygen source increases, leading to chronic hypoxia as oxygen diffusion becomes insufficient to meet cellular needs. In this phase, HIF-1α expression decreases, establishing pimonidazole as a more precise hypoxia marker [24].

Our research findings substantiate this theory, suggesting that by around 10 days post-implantation, the tumor stabilized into chronic hypoxia, with pimonidazole, rather than HIF-1α, emerges as a reliable indicator of hypoxia, while T2 and T2* values were the promising imaging marker for chronic hypoxia *in* *vivo*.

A series of research conducted by Stadlbauer Andreas categorizes the oxygen metabolism of glioma cells into five distinct classes based on qBOLD parameters: glycolysis, oxidative phosphorylation, hypoxia with angiogenesis, hypoxia without angiogenesis, and necrosis [9, 14]. This delineation suggests that OEF and CMRO_2_ alone do not adequately indicate the hypoxic status. Our study aligns with these findings, showing no significant correlation between OEF and CMRO_2_ expressions and pimonidazole levels in tumors, indicating that these tumors predominantly exist in states of glycolysis, oxidative phosphorylation, and hypoxia accompanied by angiogenesis.

This study utilized qBOLD scanning to track oxygen metabolism in C6 gliomas, which can closely mimic the critical characteristics of human gliomas, including rapid growth, extensive angiogenesis, invasive behavior, and a hypoxic environment as well [25]. Results indicate the progressive declines in the T2 and T2* values within the tumor at 12, 15, and 18 days. Concurrently, there was a gradual reduction in perfusion, an increase in OEF values with significantly heterogeneous distribution, and a slight rise in CMRO_2_ values. These outcomes indicate a transition from glycolysis and oxidative phosphorylation towards hypoxia and necrosis, broadly aligning with Andreas’s theoretical framework but with a notable divergence: while Andreas categorizes gliomas based on discrete metabolic states, our data suggest these states are part of a continuously evolving with tumor growth [14].

Metformin, a cornerstone medication for the treatment of type 2 diabetes, has been identified as a potential adjuvant therapy for cancer [15, 26]. One of the anticancer mechanisms of metformin is predicated upon the alteration of the equilibrium between the activation of adenosine monophosphate-activated protein kinase and the inhibition of the mammalian target of rapamycin, which can lead to the negative regulation of HIF-1α and vascular endothelial growth factor, thus ameliorating tumor hypoxia [27]. In this research, T2 and T2* parameters within C6 tumors were maintained at higher levels by day 15, and OEF together CMRO_2_ values remained consistently lower in the treatment group, suggesting that metformin may have a role in delaying the progression of tumor hypoxia. Despite the lack of positive outcomes from Phase II clinical trials of metformin combined with temozolomide in glioma chemotherapy, the findings could assist in identifying a more suitable patient demographic for metformin treatment [28].

The study bears certain limitations. Firstly, as it is an animal study with a relatively small sample size, the results may be prone to certain biases. Secondly, the utilization of the rat glioma C6 cell line, which differs from human gliomas, may limit the direct applicability of the findings. Future research could benefit from incorporating human-derived gliomas to bridge this gap.

In conclusion, T2 and T2* parameters in qBOLD imaging served as indicators of the level of chronic hypoxia within a tumor, while oxygen metabolism parameters including OEF and CMRO_2_ provided insights into the metabolic state of oxygen in tumor cells. The oxygen metabolic state in gliomas appears as a continuously evolving process, and metformin may have the capacity to decelerate the progression of hypoxia within the tumor to a certain extent.

## Supplementary information

ELECTRONIC SUPPLEMENTARY MATERIAL

## Data Availability

The datasets generated and/or analyzed during the current study are not publicly available due to ethical restrictions but are available from the corresponding author upon reasonable request.

## Abbreviations

CBF: Cerebral blood flow
CBV: Cerebral blood volume
CMRO_2_: Cerebral metabolism rate of O_2_
DSC: Dynamic susceptibility contrast
GB: Glioblastoma
GM: Gray matter
HIF-1*α*: Hypoxia inducible factor-1*α*
NAWM: Normal-appearing white matter
OEF: Oxygen extraction fraction
qBOLD: Quantitative blood oxygen level-dependent
ROI: Region of interest
WM: White matter
