# Supplementary material for: Assessment of hypoxia and its dynamic evolution in glioblastoma via qBOLD MRI: a comparative study with metformin treatment
Source: Eur Radiol Exp. 2024 Dec 2;8:134. doi: 10.1186/s41747-024-00533-2 (PMC11612089; doi:10.1186/s41747-024-00533-2)
Supplement: Supplementary file 1 — ELECTRONIC SUPPLEMENTARY MATERIAL [file 41747_2024_533_MOESM1_ESM.pdf]

Assessment of hypoxia and its dynamic evolution in glioblastoma via qBOLD MRI: a comparative study with metformin treatment

ELECTRONIC SUPPLEMENTARY MATERIAL

Supplementary 1. The detailed construction procedure for tumor-bearing rats

Male Wistar rats (age 6–10 weeks, 250–300 g) were anesthetized by intravenous injection of medetomidine (0.4 mg/kg) and ketamine (70 mg/kg). Body temperature was maintained at around 37.5℃. Under stereotactic guidance, a 1 mm diameter hole was drilled in the skull for injection of approximately 1×10^6 C6 glioma cells in 5μl PBS solution into the basal ganglia (3 mm left of the bregma at a 4.5 mm depth in the brain) over 6 minutes. Postoperative pain was managed with Metamizol (50 mg/kg) and rats were monitored for mood behavior and activity levels via dropping volume as a surrogate measurement.

Supplementary 2. The detailed parameters for the above-mentioned sequences and the specifics regarding post-processing of qBOLD data

Anatomical T1WI were acquired by a 2D fast spin-echo (FSE) sequence with following parameters: repetition time (TR) = 2500 ms; echo time (TE) = 25 ms; slice thickness = 1 mm; field of view (FOV) = 60 × 60 mm^2, matrix size = 256 × 256. Anatomical T2WI were also obtained using a 2D FSE sequence with: TR = 4500 ms; TE = 110 ms; FOV = 60 × 60 mm^2; matrix size = 256×256; slice thickness = 1 mm. Following the scanning, tumor volumes were calculated. All healthy rats and C6-bearing rats with tumor volume

reached 50 mm^3 received the subsequent qBOLD MRI examination. The qBOLD MRI protocol included three distinct sequences: T2 Mapping, T2* Mapping, and Dynamic Susceptibility Contrast (DSC) MRI. T2 Mapping was conducted using a 2D multi-echo spin-echo sequence (TR = 1500 ms; TE = 12.5/25/37.5/50/62.5/75/87.5/100 ms; FOV = 30 × 30 mm^2; matrix size = 256×256; slice thickness = 1 mm; NEX = 2). T2* Mapping was executed with a 3D multi-echo gradient-echo sequence (TR = 100 ms; TE = 3/9/15/21/27/33 ms; FOV = 30 × 30 mm^2; matrix size = 256×256; slice thickness = 1 mm; NEX = 2). The DSC MRI was performed using a gradient-echo echo-planar imaging (EPI) sequence (TR = 400 ms; TE = 7.3 ms; FOV = 30 × 30 mm^2; matrix size = 128 × 128; slice thickness = 2 mm) with an injection of a suspension of 0.6 ml Gd-DTPA (Magnevist, Schering, Berlin, Germany) followed by 0.5 ml NaCl in a high-pressure syringe into the tail vein with a flow rate of 2 ml/s after the initial 10 phases. The total scan time of qBOLD was approximately 18 minutes. Post-scanning, tumor-bearing rats that underwent successful qBOLD scannings were sacrificed for immunochemistry study.

The qBOLD data was post-processed using Functool software on an Advantage Workstation (Version 4.6; GE Healthcare, Milwaukee, WI, USA) and Matlab (MathWorks Inc., Natick, MA). CBV maps and CBF maps were calculated via Brainstat AIF function on AW workstation. Then, MR_SO_2 maps and OEF maps were calculated according to the quantitative BOLD approach described in previous studies[14] using:

$$(1 - MR\_SO_2) = (R_2{}^* - R_2)/(\frac{4}{3} \times CBV \times \gamma\Delta\chi \times Hct \times B_0);$$

$$OEF = \left(1 - MR_{SO_2}\right) \times 100\%$$

where Hct=0.42×0.85 is the microvascular hematocrit fraction and $\Delta\chi$=0.264×10^{-6} is the difference between the magnetic susceptibilities of fully oxygenated and fully deoxygenated haemoglobin. γ= 2.67502×10^{8} rad/s/T is the nuclear gyromagnetic ratio.

MR_CMRO2 maps in $\mu mol/100g/min$ were then obtained using:

$$MR\_CMRO_2 = (1 - MR\_SO_2) \times CBF \times Ca$$

Where Ca=8.68$\mu mol/mL$ is the arterial blood oxygen content.
